# Future trends of water resources and influences on agriculture in China

**DOI:** 10.1371/journal.pone.0231671

**Published:** 2020-04-17

**Authors:** Jincai Zhao, Zheng Wang

**Affiliations:** 1 School of Business, Henan Normal University, Xinxiang, Henan, China; 2 Key Laboratory of Geospatial Technology for the Middle and Lower Yellow River Regions (Henan University), Ministry of Education, Kaifeng, China; 3 College of Environment and Planning, Henan University, Kaifeng, Henan, China; 4 Key Laboratory of Geographic Information Science, Ministry of Education, East China Normal University, Shanghai, China; Harran University, TURKEY

## Abstract

Water resources are indispensable for all social-economic activities and ecosystem functions. In addition, changes in water resources have great significance for agricultural production. This paper uses five global climate models from CMIP5 to evaluate the future spatiotemporal variation in water resources in China under four RCP scenarios. The results show that the available precipitation significantly decreases due to evapotranspiration. Comparing the four RCP scenarios, the national average of the available precipitation is the highest under the RCP 2.6 and 4.5 scenarios, followed by that under the RCP 8.5 scenario. In terms of spatial distribution, the amount of available precipitation shows a decreasing trend from southeast to northwest. Regarding temporal changes, the available precipitation under RCP 8.5 exhibits a trend of first increasing and then decreasing, while the available precipitation under the RCP 6.0 scenario exhibits a trend of first decreasing and then increasing. Under the RCP 2.6 and 4.5 scenarios, the available precipitation increases, and the RCP 4.5 scenario has a higher rate of increase than that of RCP 2.6. In the context of climate change, changes in water resources and temperature cause widespread increases in potential agricultural productivity around Hu’s line, especially in southwestern China. However, the potential agricultural productivity decreases in a large area of southeastern China. Hu’s line has a partial breakthrough in the locking of agriculture, mainly in eastern Tibet, western Sichuan, northern Yunnan and northwestern Inner Mongolia. The results provide a reference for the management and deployment of future water resources and can aid in agricultural production in China.

## 1. Introduction

Water resources are a fundamental natural resource, and the variation in water resources is of great significance for the existence and development of humankind. With rapid social and economic development, water has become an important constraint for sustainable development. Moreover, with global climate change, agricultural production is affected by water resources to a great extent. Understanding the changes in water resources is not only beneficial to formulating plans for the utilization and protection of water resources but also helpful for agricultural development.

Water resource assessment has attracted the attention of scholars worldwide [1–3]. For example, Geng et al. [4] calculated the total water resources and analysed the spatial distribution and temporal variation in northwestern China by using 40 years of synchronous data of rainfall, evaporation and runoff. Ding et al. [5] explored the regional differences over western China in terms of variations in climate and discharge using annual precipitation and discharge data. Li et al. [6] analysed the changing trends and periods of renewable water resources in China during 1956–2010 based on a national water resources assessment.

Climate change is one of the most significant challenges facing our generation and the next generation[7–9]. In the context of climate change, changes in temperature and precipitation are obvious. Climate change can drive changes in the water cycle, which further affect the supply and distribution of water resources in major river basins. The impact of climate change on water resources has attracted the attention of scholars[10], and historical meteorological and hydrological data have been utilized to analyse the relationship between climate change and water resources. For instance, Chen et al. [11] analysed the impact of climate change on water resources in the Tarim River Basin during the 1950–2000 period, and this study showed that the streamflow from the headwater exhibited a significant increase during the 1980–2000 period. Zhang et al. [12] further showed that precipitation in the Tarim River Basin showed a significant increasing trend, which in turn led to increasing streamflow. Miao et al. [13] identified the characteristics of streamflow change in the Yellow River by using approximately 50 years of natural and observed streamflow data from 23 hydrological stations. It is more realistic to quantify the potential effect of future climate change on water resources.

The projected data under climate change scenarios are widely adopted to assess the future variation in water resources. Gao and Huang [14] assessed the impact of future climate change on water resources in North China and concluded that evaporation is greater than precipitation, and water deficiency is very serious and often leads to severe droughts in spring. Zhang et al. [15] predicted and analysed the amount of water resources in the Nanjing region in thirty different future climate scenarios, concluding that climate change can greatly impact water resources. Barnett et al. [16] assessed the effects of climate change on water resources in the western United States and indicated that future water demand may not be satisfied. Arnell [17] simulated river runoff under current and future climate change scenarios and assessed the pressure of climate change and population growth on future water resources on a global scale. Chen and Clarke [18] predicted the change in water resources in the Jialing River catchment in 2050 and 2100 based on different climate change scenarios. The results showed that the annual runoff will decrease by 23.0–27.9% in 2050 and 28.2–35.2% in 2100 for the disadvantaged conditions. Li et al. [19] assessed the potential impact of climate change on water resources, including precipitation, runoff, soil water content and evapotranspiration, in the Heihe watershed under A2, B2 and GGa scenarios. On this basis, Li et al. [20] utilized the CA-Markov model to develop land use scenarios and discussed the role of land use change in the response of water resources to future climate change. Hao et al. [21] demonstrated that the warming and drying trend of climate change in the past 50 years resulted in a significant reduction in the total amounts of water resources in Hebei Province, and in the future, the increased precipitation and temperature will cause the total amounts of water resources to increase. Sun et al. [22] used the projected precipitation and temperature data in Jinjiang Basin, China, under the A1B emission scenario and showed that runoff in summer to early autumn exhibits an increasing trend, while during the rest of the year, runoff shows a decreasing trend, especially in the spring season. Kopytkovskiy et al. [23] utilized future climate change models to drive hydrologic models and evaluate the future water resources and hydropower potential of the Upper Colorado River Basin. Mourato et al. [24] assessed the impacts of different climate change scenarios on water availability in Mediterranean watersheds using the SHETRAN hydrological model. Devkota and Gyawali [25], based on the IPCC-SRES A1B scenario, assessed the hydrological regime of the Koshi River Basin in Nepal under climate change. Based on 43 projection results of the IPCC GG, GS, A2 and B2 scenarios, Wang and Zhang [26] predicted that from 2000, both the temperature and precipitation in China will rise in the next 50 years, and runoff in the main river basins will also increase.

Agriculture is the sector most strongly affected by climate change. Climate change alters the light, temperature, and water in the process of crop growth and development, which directly affects the potential productivity of crops. To explore the effect of climate conditions on potential agricultural productivity from the perspective of economics, climate factors, such as temperature and precipitation, should be considered [27]. Theoretically, potential agricultural productivity is positively related to temperature and sunshine hours. Precipitation is beneficial to crop growth within a certain range, while it is not conducive to crop growth beyond the appropriate range. Among the factors linked to climate change, the impact of precipitation on agriculture is considered to be the most important [28].

Many studies have been conducted to quantify the influences of climate change on agriculture. For example, Chavas et al. [29] examined the potential impacts of climate change on the productivity of five major crops in eastern China. Gornall et al. [30] analysed the possible impact of climate change on the net primary production potential of global agricultural land during 2020–2050. Lobell et al. [31–32] discussed the effects of climate change on global crop production during 1980–2008. Yuan et al. [33] analysed and predicted the change in the agricultural climate resources and the effects of climate change on the variety distribution and climatic potential productivity of spring maize from 1951 to 2100 under the future A1B climatic scenario in Northeast China. Baldos and Hertel [34] examined how agricultural productivity and climate change affect the future of global food security, and the results showed that global food security has improved in 2006–2050, mainly due to the growth in agricultural productivity.

Research on the impact of climate change on regional water resources and agricultural productivity has yielded rich results. The fifth IPCC report proposed new scenarios for carbon emissions. The current literature includes few assessments of water resources under the new climate change scenario. How will water resources and potential agricultural productivity change in the future? It is important to clarify the impact of climate change on water resources and potential agricultural productivity, which is related to ecological functions, agricultural production, and human survival and development. Based on the latest four climate change scenarios (RCP 2.6, 4.5, 6.0 and 8.5), this paper considers both precipitation and evapotranspiration and analyses the variation in the available precipitation and its influences on agricultural productivity under different climate change scenarios.

## 2. Materials and methods

### 2.1 Data

This study employed the average value of five GCMs, namely, MIROC-ESM-CHEM, NorES1-M, IPSL-CM5A-LR, GFDL-ESM2M and HadGEM2-ES, which are all derived from the Inter-Sectoral Impact Model Intercomparison Project (ISI-MIP, https://www.isimip.org/). These datasets were interpolated to a 0.5°×0.5° latitude-longitude grid in space, and bias correction was performed. Hempel et al. [35] provided the detailed processing procedure. The projected data are provided under four RCP climatic scenarios (including RCP 2.6, RCP 4.5, RCP 6.0 and RCP 8.5). The time coverage is from 2006 to 2099 at daily time steps. The variables involved in this study include daily average temperature, daily maximum temperature, daily minimum temperature, precipitation, relative humidity and wind speed. To verify the accuracy of the projected climatic data, historical observational data from 2006–2016 were utilized, which were provided by the Climatic Data Center, National Meteorological Information Center, China Meteorological Administration (http://data.cma.cn).

### 2.2 Calculation of reference evapotranspiration

The Penman-Monteith formula recommended by the Food and Agriculture Organization (FAO) of the United Nations was used to calculate the reference evapotranspiration (ET_0_), which has been widely used and proven to be of high accuracy and practicability [36–40]. The Penman-Monteith formula is as follows:

$$ET_{0}=\frac{0.408\Delta \left(R_{n}-G\right)+\gamma \frac{900}{T+273}U_{2}\left(e_{a}-e_{d}\right)}{\Delta +\gamma \left(1+0.34U_{2}\right)},$$ (1)

where *ET*_*0*_ is in millimetres per day (mm d^-1^); Δ is the saturation vapor pressure/temperature curve (kPa °C^-1^); *R*_*n*_ is the net radiation from the canopy (MJ m^-2^ d^-1^); *G* is the soil heat flux (MJ m^-2^ d^-1^); *T* is the average daily temperature (°C); *U*_*2*_ is the wind velocity (m s^-1^); *e*_*a*_ is the saturation vapor pressure (kPa); *e*_*d*_ is the actual water vapor pressure (kPa); and *γ* is the psychometric constant (kPa °C^-1^). The details are as follows.

(a) Saturation vapor pressure:

$$e_{a}=0.611\cdot \exp(\frac{17.27T}{T+237.3})$$ (2)

(b) Actual water vapor pressure:

$$e_{d}=RH/\left[\frac{50}{e_{a}(T_{\min})}+\frac{50}{e_{a}(T_{\max})}\right]$$ (3)

where *RH* is the relative humidity (%) and *T*_*max*_ and *T*_*min*_ are the daily maximum and minimum temperatures, respectively.

(c) Saturation vapor pressure/temperature curve:

$$\Delta =\frac{4098e_{a}}{{\left(T+237.3\right)}^{2}}$$ (4)

(d) Soil heat flux:

$$G=0.38\cdot (T_{d}-T_{d-1})$$ (5)

where *T*_*d*_ and *T*_*d-1*_ are the average daily temperatures on the *d*th and *d-1*th days, respectively.

(e) Psychometric constant:

γ=0.00163⋅P/λ (6)

$$P=101.3\cdot {\left(\frac{293-0.0065Z}{293}\right)}^{5.26}$$ (7)

$$\lambda =2.501-\left(2.361\times 10^{-3}\right)\cdot T$$ (8)

where *P* is the air pressure (kPa); *λ* is the latent heat (MJ·kg^-1^); and *Z* is the altitude (m).

(f) Net radiation:

$$R_{n}=R_{ns}-R_{nl}$$ (9)

where *R*_*ns*_ is the net shortwave radiation (MJ/m^2^·d), and *R*_*nl*_ is the net longwave radiation (MJ/m^2^·d). *R*_*ns*_ and *R*_*nl*_ are calculated as follows.

$$R_{ns}=0.77\cdot \left(0.19+0.38n/N\right)R_{a}$$ (10)

$$R_{nl}=2.45\times 10^{-9}\cdot \left(0.9n/N+0.1\right)\cdot \left(0.34-0.14\sqrt{e_{d}}\right)\cdot \left(T_{kx}{}^{4}+T_{kn}{}^{4}\right)$$ (11)

$$R_{a}=37.6\cdot d_{r}\cdot \left(W_{s}\cdot \sin\varphi \cdot \sin\delta +\cos\varphi \cdot \cos\delta \cdot \sin W_{s}\right)$$ (12)

$$d_{r}=1+0.033\cdot \cos\left(\frac{2\pi }{365}J\right)$$ (13)

N=7.64⋅arccos(−tanφ⋅tanδ) (14)

$$\delta =0.409\cdot \sin\left(\frac{2\pi }{365}J-1.39\right)$$ (15)

where *n* is the actual sunshine duration; *N* is the theoretical maximum sunshine duration (h); R_a_ is the solar radiation at the edge of the atmosphere (MJ/m^2^·d); d_r_ is the relative distance between the sun and the earth; δ is the daily angle (rad); φ is the latitude (rad); T_ks_ and T_kn_ are the maximum and minimum absolute temperature, respectively (K); and J is the day series (January 1 is assigned to 1, added day by day).

### 2.3 Calculation of the available precipitation

The available precipitation is defined as the difference between the actual precipitation and actual evapotranspiration:

Pra=Pr−ET, (16)

where *Pra* is the available precipitation, *Pr* is the precipitation, and *ET* is the actual evapotranspiration.

The actual evapotranspiration is difficult to estimate and predict [41]. Budyko [42] proposed a relationship between the evapotranspiration ratio and potential evapotranspiration ratio on the basis of the Schreiber formula [43] and Ol’dekop formula [44]. Subsequently, researchers developed various non-parametric mathematical equations based on the functional forms of Budyko-type curves to evaluate the water balance over the long term [45–47]. As one of the best-known classical studies, the Schreiber empirical formula was still verified to be credible for China [48–49]. Therefore, this study employed the Schreiber formula to estimate the actual evapotranspiration in the future:

$$ET=Pr\left(1-e^{-\frac{ET_{0}}{Pr}}\right),$$ (17)

where *ET* is the actual evapotranspiration, and *ET*_*0*_ is the reference evapotranspiration, indicating the power of evapotranspiration.

### 2.4 Calculation of the potential agricultural productivity

As early as the 1950s, Huang [50] studied the relationships between the potential agricultural productivity and the accumulated temperature, precipitation and other climatic factors, which has been widely adopted [27,51]. In this study, the method is used to explore the effect of changes in water and temperature on the potential agricultural productivity. The calculation model, including the four elements of “light, temperature, water and soil”, is defined as:

P(Q,T,W,S)=F(Q)⋅F(T)⋅F(W)⋅F(S) (18)

where *F*(*Q*,*T*,*W*,*S*) is the potential agricultural productivity (kg/hm^2^); *F*(*Q*) is the photosynthetic potential productivity; and *F*(*T*), *F*(*W*) and *F*(*S*) are the effective coefficients of temperature, moisture and soil, respectively.

*F*(*Q*), *F*(*T*) and *F*(*W*) are calculated as follows.

F(Q)=0.123Q (19)

$$Q=Q_{0}(0.248+0.752S)$$ (20)

$$F(T)=\sum_{T>10}T$$ (21)

F(W)=Pr/ET (22)

where *Q* is the total solar radiation; *Q*_*0*_ is the latitude; *S* is the ratio of actual sunshine duration to theoretical sunshine duration; *T* is the average daily temperature; *Pr* is the precipitation; and *ET* is the actual evapotranspiration.

*F*(*S*) was obtained by using the classic soil evaluation method proposed by Leng [52], who, based on the 8 kinds of soil elements (including soil texture, pH value, nitrogen, phosphorus, potassium, organic matter content, erosion status and salinization degree), took into account the influence of the terrain altitude to evaluate the regional soil effective coefficient.

In this study, photosynthetic potential productivity does not vary with time, since it is less affected by climate change. Additionally, due to the long-term nature of soil development, the change in the soil effective coefficient under climate change is ignored. Precipitation and temperature are the variables that cause changes in potential agricultural productivity.

### 2.5 Validation of the data

#### 2.5.1 Precipitation

In this study, we employed 5 mainstream models from CMIP5: MIROC-ESM-CHEM, NorES1-M, IPSL-CM5A-LR, GFDL-ESM2M and HadGEM2-ES [53]. The data produced by the five models from ISI-MIP were used widely [54–56]. To validate the data accuracy, the overlap of the observation data and simulated data during 2006–2016 was utilized.

From the perspective of future greenhouse gas emission trends, emissions will peak in 2040 and stabilize in 2080 under RCP 4.5 [57], which is consistent with future development trends in China [58]. Therefore, the precipitation under RCP 4.5, the medium radiation force level, was selected for comparison with the actual precipitation.

**Fig 1:**
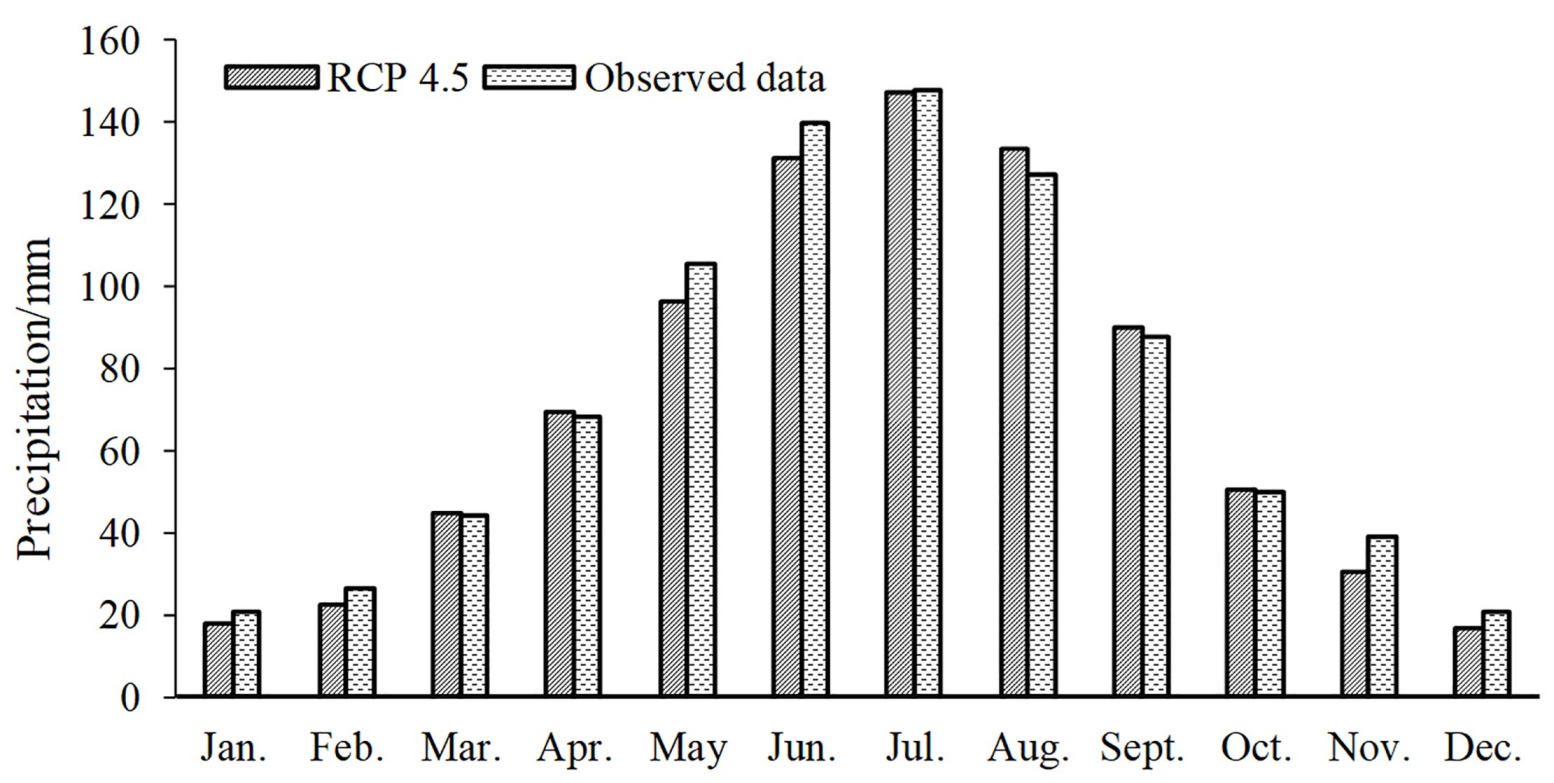
Relationship of the estimated and observed monthly precipitation.

In terms of annual precipitation, the observed average precipitation of stations is 877.9 mm yr^-1^, and the simulated average precipitation under the RCP 4.5 scenario is 850.54 mm yr^-1^, with a relative error of -3.12%. From the point of view of monthly precipitation (Fig 1), the gaps in the monthly precipitation in May, June and November are somewhat large, with differences of 9.13 mm yr^-1^, 8.77 mm yr^-1^ and 8.34 mm yr^-1^, respectively. The gaps in other months are small, especially in March, April, and October, where the simulated values are substantially equal to the observed values. According to the regression through the origin model, the regression coefficient between the observed value and the simulated value is 1.02, and R^2^ is 0.988, which has satisfactory goodness of fit. The precipitation between the simulated and observed values in each year also showed a good fit (Table 1). All the goodness of fit values were greater than 0.74, and the majority exceeded 0.8.

**Table 1:** Goodness of fit between the observed and simulated precipitation.

Years	2006	2007	2008	2009	2010	2011	2012	2013	2014	2015	2016
**R**^**2**^	0.791	0.8015	0.8463	0.8413	0.8522	0.7675	0.8488	0.7447	0.7525	0.7988	0.8476

#### 2.5.2 Available precipitation

For the validation of available precipitation, the concept of runoff depth is used. The annual runoff depth refers to the total annual runoff through a certain section of a river in one year divided by the basin area above the section. For many years, on average, the annual runoff depth is equal to the difference between the annual precipitation and annual evapotranspiration.

**Fig 2:**
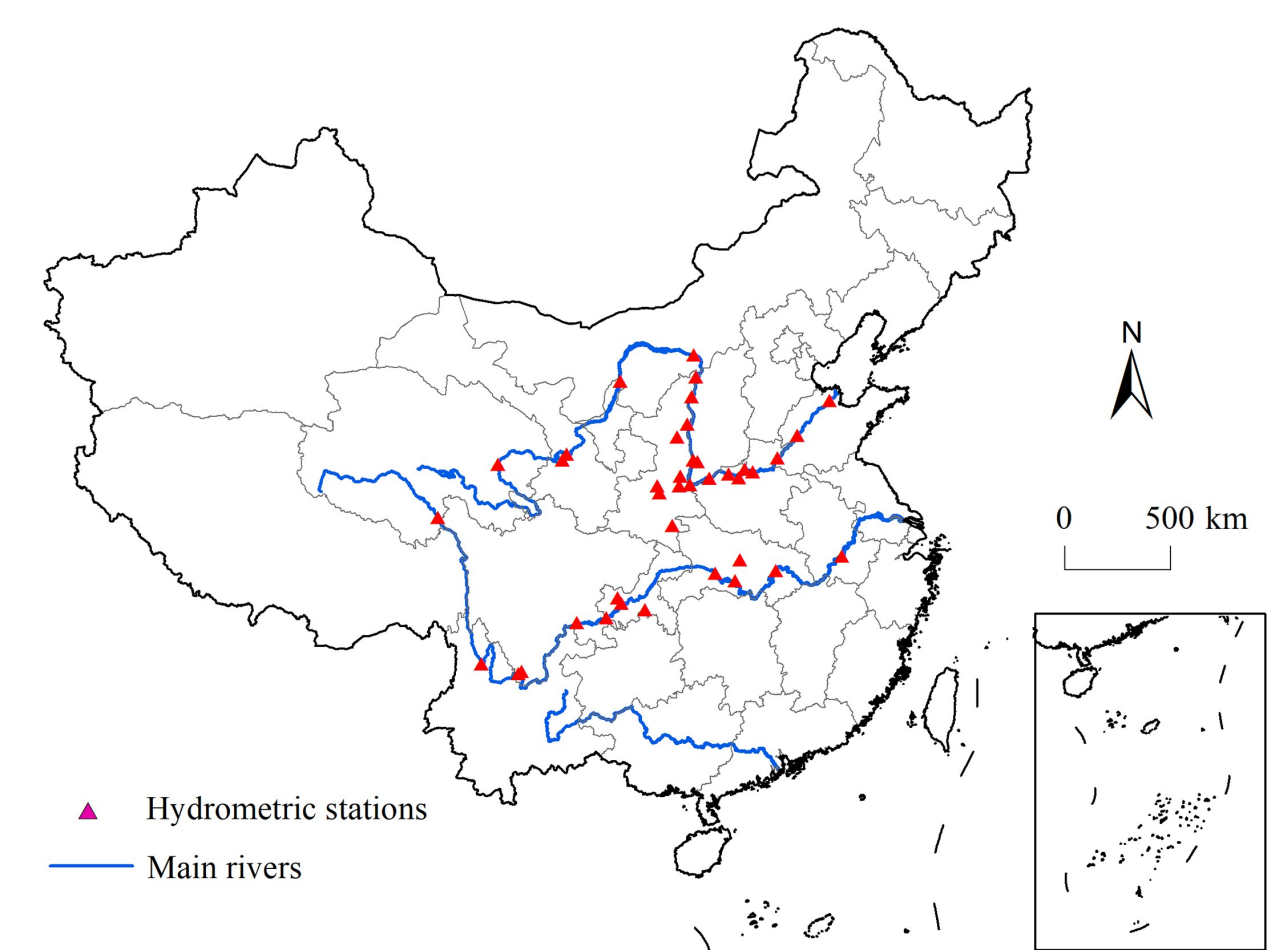
Maps of the main hydrometric stations in the Yangtze River and Yellow River.

**Fig 3:**
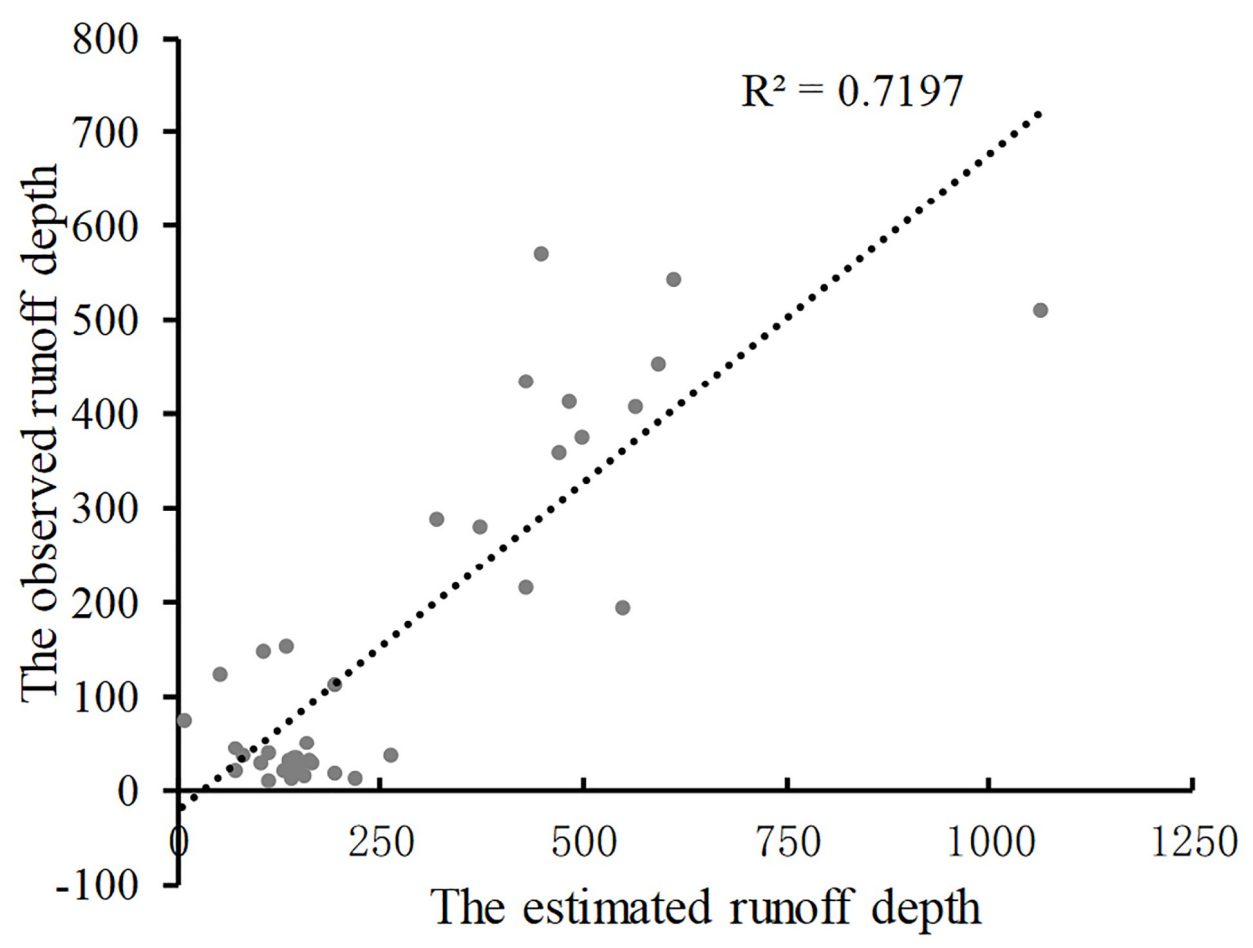
Relationship of the observed and estimated runoff depth.

The actual observed runoff depths of the hydrological stations distributed in the mainstream and tributaries of the Yangtze and Yellow rivers (Fig 2) are adopted to model the estimated runoff depth, namely, the available precipitation. The goodness of fit of the linear regression model is 0.7197 (Fig 3), which is significant at the 0.01 level. This result indicates that the estimated available precipitation has a certain degree of credibility, and its change in the future is representative.

## 3. Results

### 3.1 Precipitation

**Fig 4:**
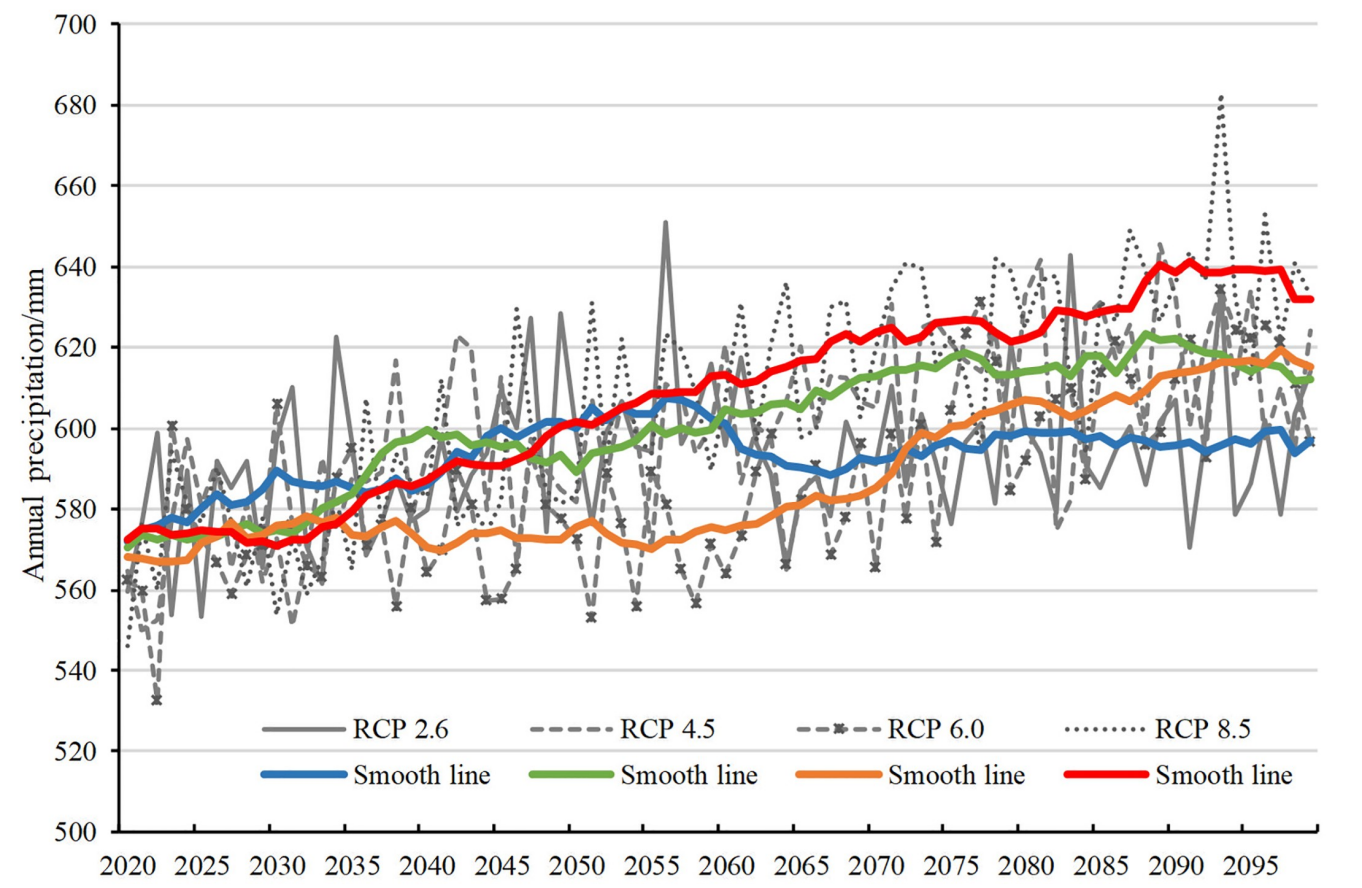
Average annual precipitation in China under the four RCPs.

Fig 4 shows the temporal variation in the average annual precipitation in mainland China. The average annual precipitation fluctuates between 2020 and 2099. Under RCP 2.6, the annual precipitation can be divided into three stages. Before 2056, the annual precipitation shows an upward trend, increasing from 571 mm to 607 mm, then begins to decrease, and after falling to 588 mm in 2067, it gradually becomes stable. Under RCP 4.5, the annual precipitation shows an overall growth trend. During the period of 2035–2050, there is a rapid increase and decrease, and a small peak appears in 2040, reaching 599 mm. Under RCP 6.0, the change in the annual precipitation is divided into two phases by the year 2065. The early stage is a steady state, and the latter stage is a rapid increase stage. The annual precipitation obviously increases under RCP 8.5. After 2056, the annual precipitation is significantly higher than that of the other three scenarios.

To analyse the trend of change, the whole study period is divided into four periods: 2010s (2006–2019), 2030s (2020–2049), 2060s (2050–2079) and 2090s (2080–2099).

**Fig 5:**
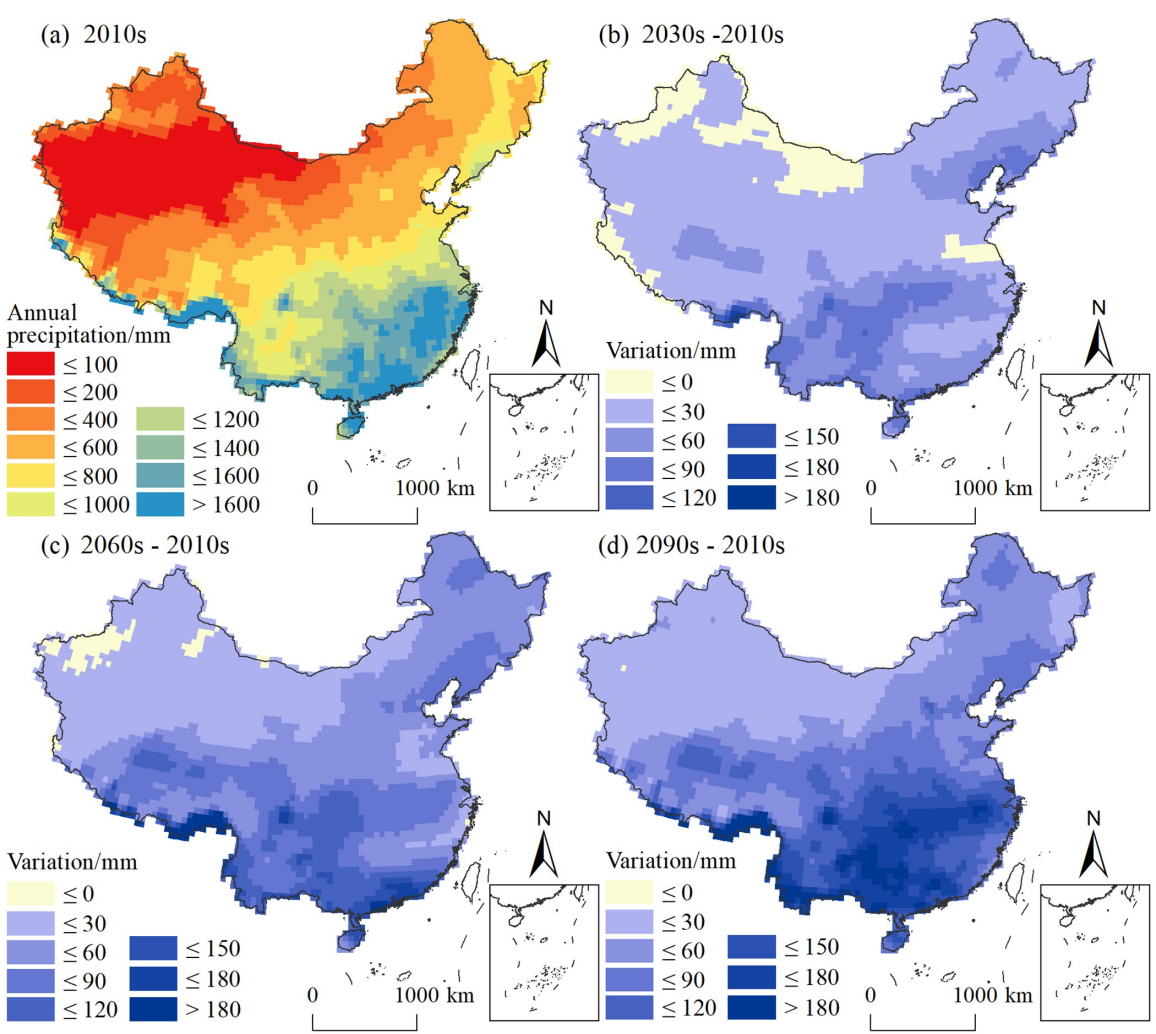
Temporal variations in the average annual precipitation under RCP 4.5.

Fig 5 compares the changes in the precipitation over the four time periods, taking the RCP 4.5 scenario as an example. The spatial distribution of precipitation is regressive from the southeastern coast to the northwestern inland.

For the temporal variation between the 2010s and 2030s, most regions show an increasing trend of precipitation. For example, the southwestern region, central southern region and the Bohai Rim region show obvious humidification. The areas characterized by aridification are mainly located in the northwestern region, such as western Inner Mongolia and northern Xinjiang.

In the 2060s, the annual precipitation further increases. The increase in annual precipitation exceeds 30 mm in most areas. The annual precipitation increases by 60 mm in most areas of Tibet, Sichuan, Chongqing, Yunnan, Guizhou, Guangxi and Guangdong, and the increase in some areas even exceeds 120 mm. In northwestern China, the increase in precipitation is small, and parts of northern Xinjiang show a downward trend in precipitation.

By the 2090s, almost all of the country shows an increasing trend in annual precipitation. Especially in the southern region, the increase is mainly above 60 mm. The change in the annual precipitation in the northwestern region is relatively small, with an increase of less than 30 mm.

### 3.2 Evapotranspiration

**Fig 6:**
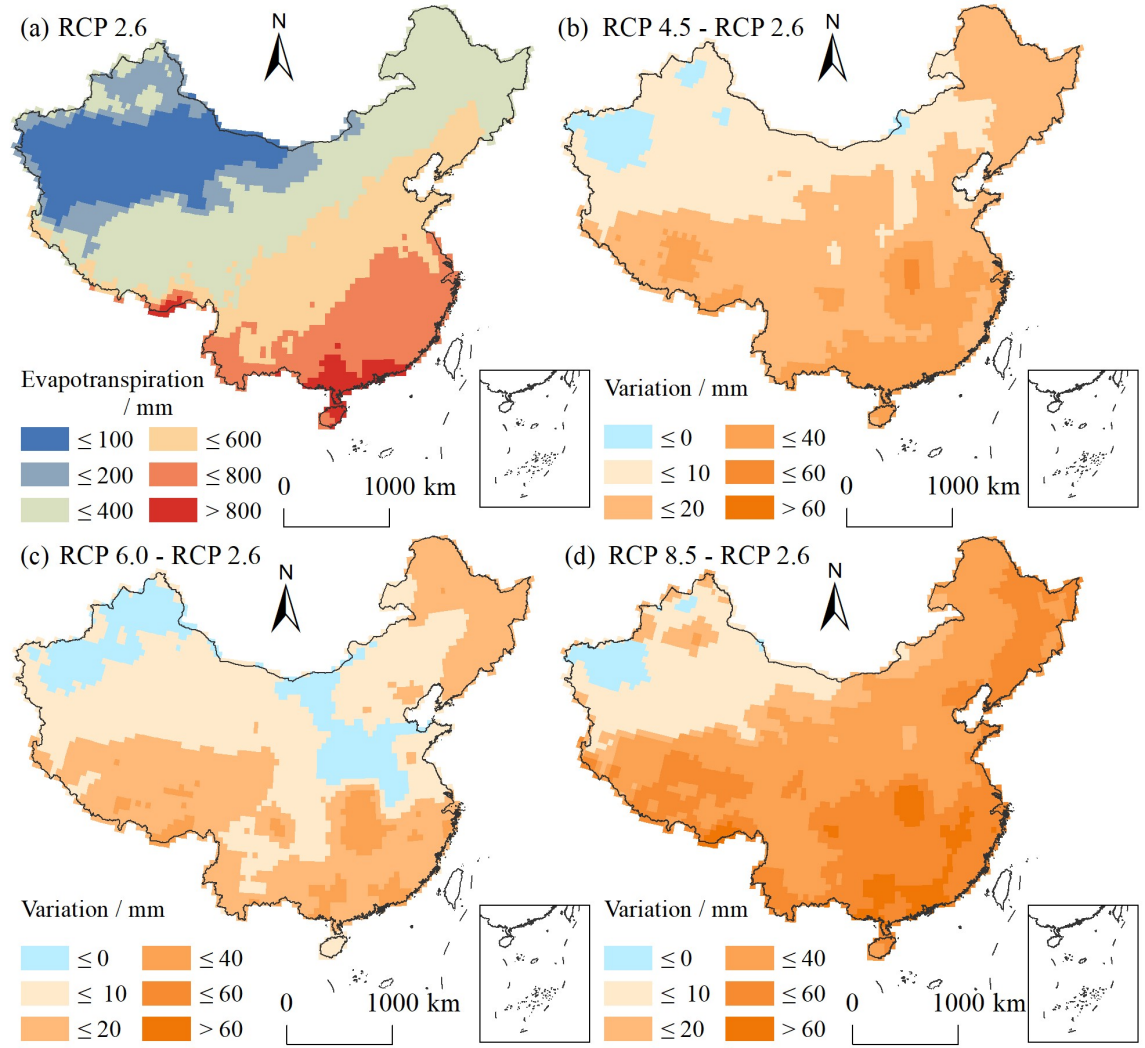
Changes in evapotranspiration under RCP scenarios.

Fig 6(A) shows that the spatial variation in evapotranspiration is consistent with that of precipitation, decreasing from southeast to northwest. Evapotranspiration varies slightly under different RCP scenarios (Fig 6B–6D). Among these scenarios, a significant increase in evapotranspiration appears under the RCP 8.5 scenario. The spatial characteristics of evapotranspiration variation in China show greater increases in the southeast and smaller increases in the northwest.

**Fig 7:**
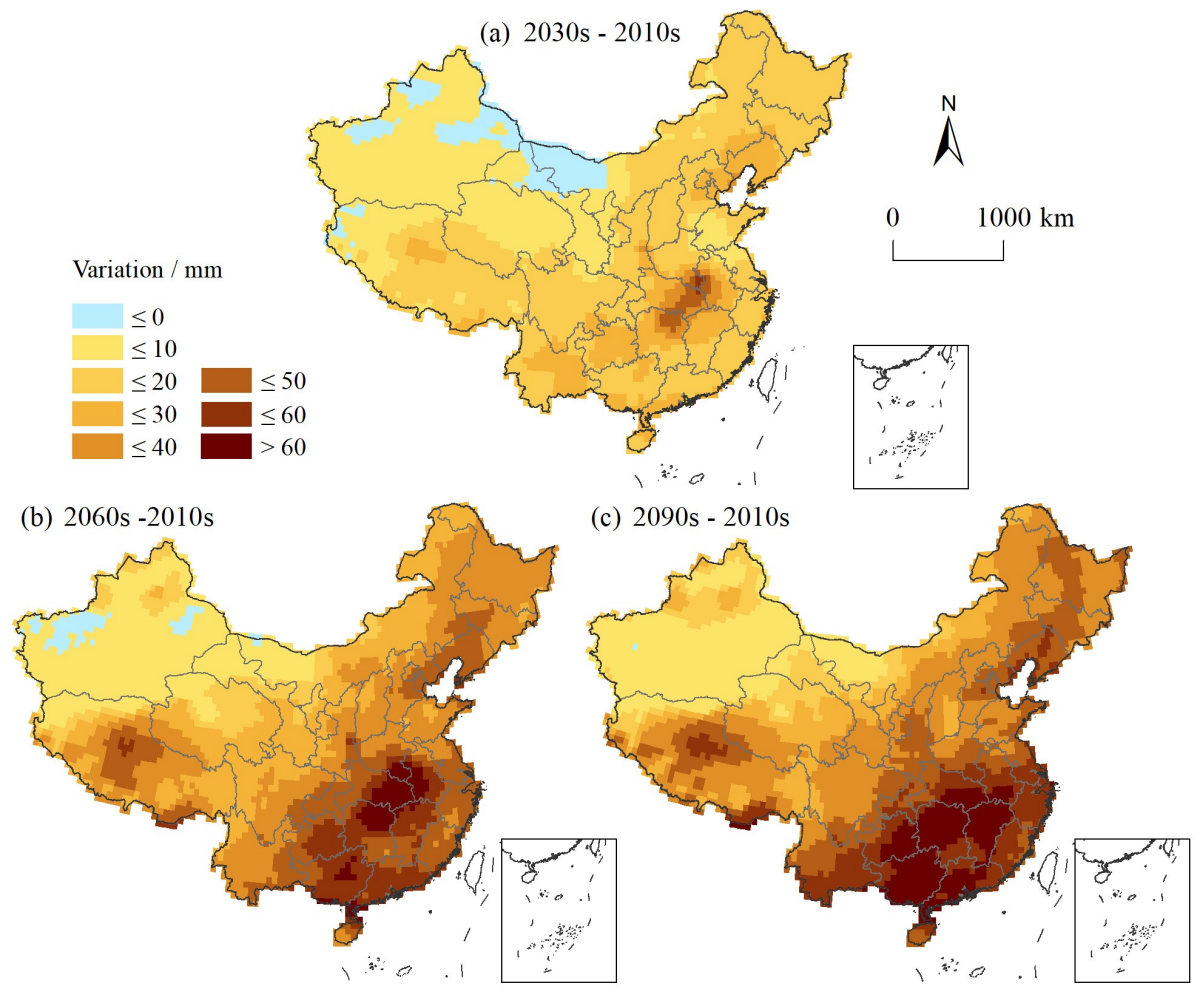
Evapotranspiration during different periods under RCP 4.5.

Fig 7 shows the temporal variation in evapotranspiration under the RCP 4.5 scenario, with the 2010s as a baseline. Overall, the increasing trend of evapotranspiration is the main characteristic for the whole country, and with the passage of time, the evapotranspiration intensity increases. Spatially, evapotranspiration in the southeast region is high, and the increase in evapotranspiration is also higher.

### 3.3 Available precipitation

**Fig 8:**
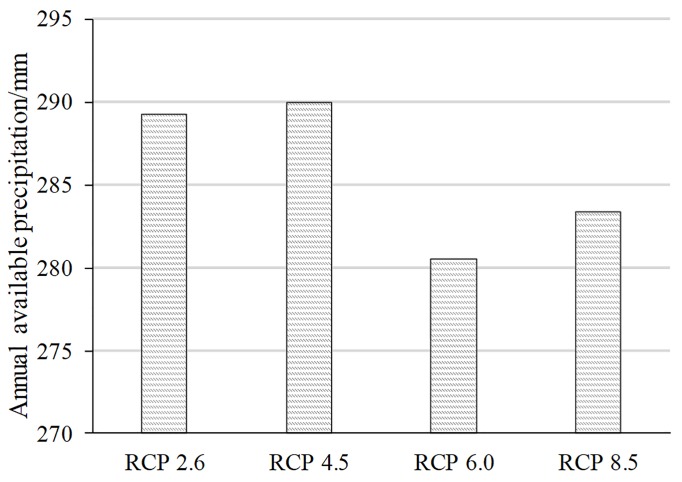
Average annual available precipitation in China under different scenarios.

Under the combined action of precipitation and evapotranspiration, the available precipitation is significantly lower than the precipitation. The difference in the available precipitation under different scenarios is obvious (Fig 8). Regarding the national average, the available precipitation under RCP 6.0 is the smallest because the precipitation is relatively small while the evapotranspiration significantly increases. The precipitation and evapotranspiration under RCP 8.5 are relatively high, which results in low available precipitation. The available precipitation is high under the RCP 2.6 and 4.5 scenarios, among which the available precipitation under RCP 4.5 is slightly higher than that under RCP 2.6.

**Fig 9:**
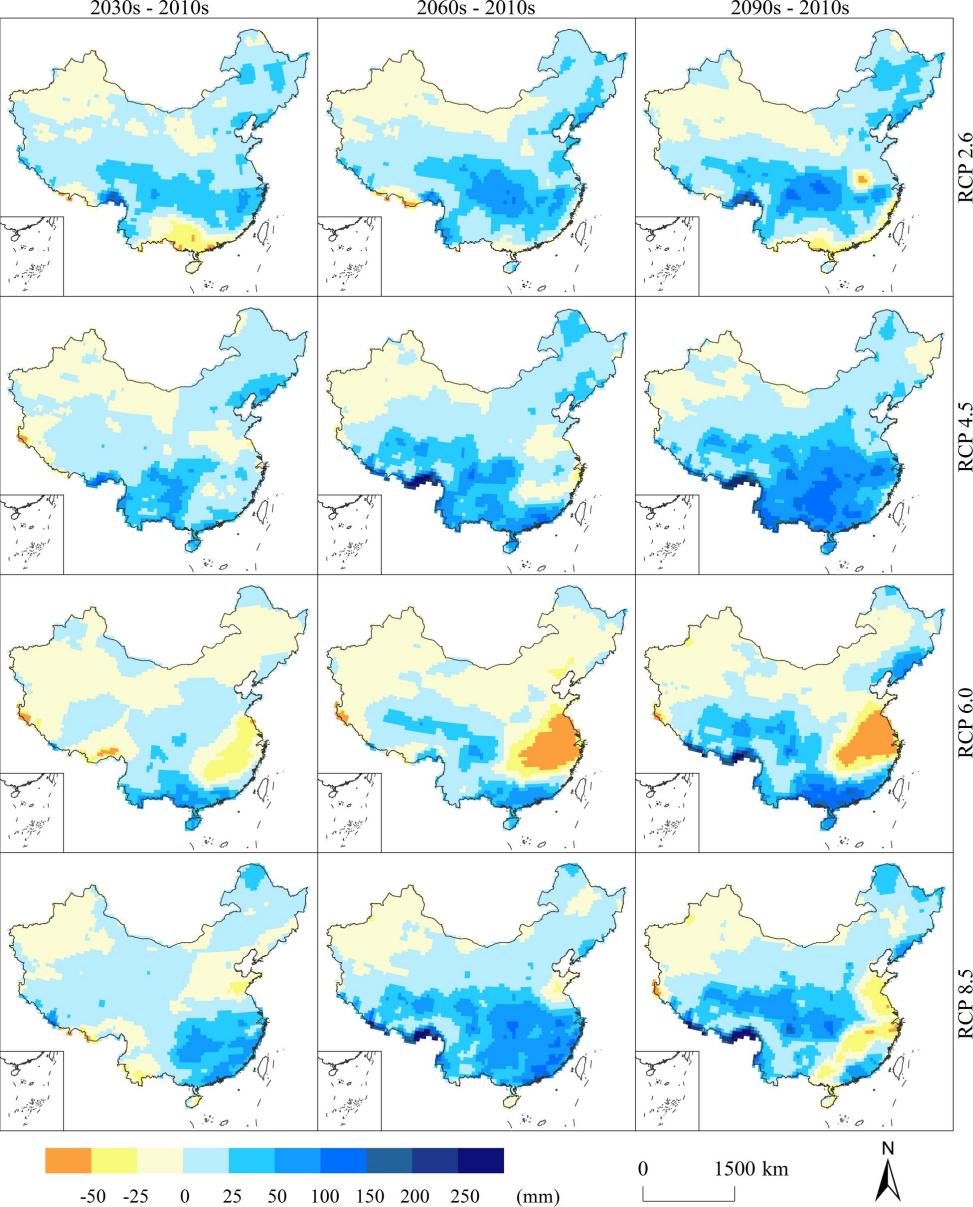
Changes in the average annual available precipitation under RCP scenarios.

The temporal variation in the available precipitation varies widely in space (Fig 9). Under RCP 2.6, the available precipitation is mainly increased. The regions with substantial increases in the 2030s are mainly located in Sichuan, Chongqing, Guizhou, Jiangxi, Zhejiang and Fujian, and the increase amplitude is generally above 25 mm. Areas with a large decline are found in Guangxi and western Guangdong, with a decrease of more than 25 mm. In addition, most of Xinjiang shows a downward trend in the available precipitation with a decrease of less than 25 mm. In the 2060s, the spatial extent of the decrease in available precipitation in northwestern China expands, including large areas in Xinjiang, northwestern Gansu and western Inner Mongolia. The available precipitation in the Sichuan-Chongqing region increases by more than 50 mm. The humidification trend is obvious in the southern region. In the 2090s, the arid area of the northwestern region extends to the southeast. The aridification trend is obvious in the southeastern coastal area and the at junction of Henan and Anhui, while the degree of humidification in the Sichuan-Chongqing region increases, indicating that the available precipitation becomes more uneven over time.

Under RCP 4.5, the amount of available precipitation also shows an increasing trend. In the 2030s, the areas with an increase of more than 25 mm are mainly distributed in southeastern Sichuan, Chongqing, western Hubei, Guizhou, Yunnan, western Guangxi, southern Guangdong, and Liaoning. Areas with a downward trend are mainly located in Xinjiang in the northwest, western Inner Mongolia, western Tibet, Henan, northern Anhui, and northern Jiangsu. In the 2060s, areas undergoing aridification narrow in the northwestern region. The North China Plain still shows an aridity trend, and the spatial extent of drought in Zhejiang, Jiangxi, and Hunan expands. The increase in the available precipitation in northwestern and southern China is obvious, with an increase of more than 50 mm in most areas. By the 2090s, the increase in the available precipitation exceeds 50 mm in almost the entire southern region, and even the increase in Guizhou and Guangxi exceeds 100 mm. Areas of aridification further decrease in the northwestern region. Overall, the available precipitation increases over time.

Under RCP 6.0, the available precipitation shows a downward trend in the middle and lower reaches of the Yangtze River and an upward trend in the southwestern and southern regions. In the 2030s, approximately half of the country exhibits a downward trend in the available precipitation, mainly in the northwestern, northeastern, and southern parts of central and eastern China. The available precipitation in southern China is on the rise, and the increase is more than 25 mm. In the 2060s, areas with downward trends noticeably expand, covering almost all of northern, central and eastern China. Among these regions, an obvious decline appears in central and eastern China with a drop of more than 50 mm. South China still shows an upward trend. In the 2090s, areas with a downward trend decrease. The northeastern region has changed from a downward trend to an upward trend. Although areas with downward trends in central and eastern China decrease, the decline in the available precipitation increases. The available precipitation in the Sichuan-Tibet and South China regions obviously increases. The results show that the spatial differentiation increases.

Under RCP 8.5, the available precipitation mainly shows an increasing trend. In the 2030s, the increasing trend is obvious in southeastern China, with an increase above 25 mm. Northwestern China, northern China and Yunnan mainly show a downward trend, and the decline amplitude is mainly less than 25 mm. In the 2060s, the available precipitation further increases. Most of the southern regions show an upward trend with an increase of more than 25 mm. In the 2090s, areas with downward trends expand greatly. The available precipitation decreases in eastern China and the provinces of Guizhou and Guangxi. The areas where the available precipitation increases significantly are mainly located in the Sichuan-Chongqing region, the Qinghai-Tibet border, and Guangdong with increases of more than 50 mm. In summary, the available precipitation mainly shows an upward trend in the early stage and then a downward trend. The reason is likely that the evapotranspiration greatly increases over time, and the precipitation does not receive enough supply. As a result, the available precipitation decreases in most areas.

### 3.4 Potential agricultural productivity

Changes in precipitation, coupled with rising temperatures in the context of climate change, are likely to affect changes in potential agricultural productivity. According to the four elements of “light, temperature, water and soil”, the potential agricultural productivity in the 2060s under the RCP 4.5 and 8.5 scenarios is calculated and compared with that of the period of 1980–2000.

**Fig 10:**
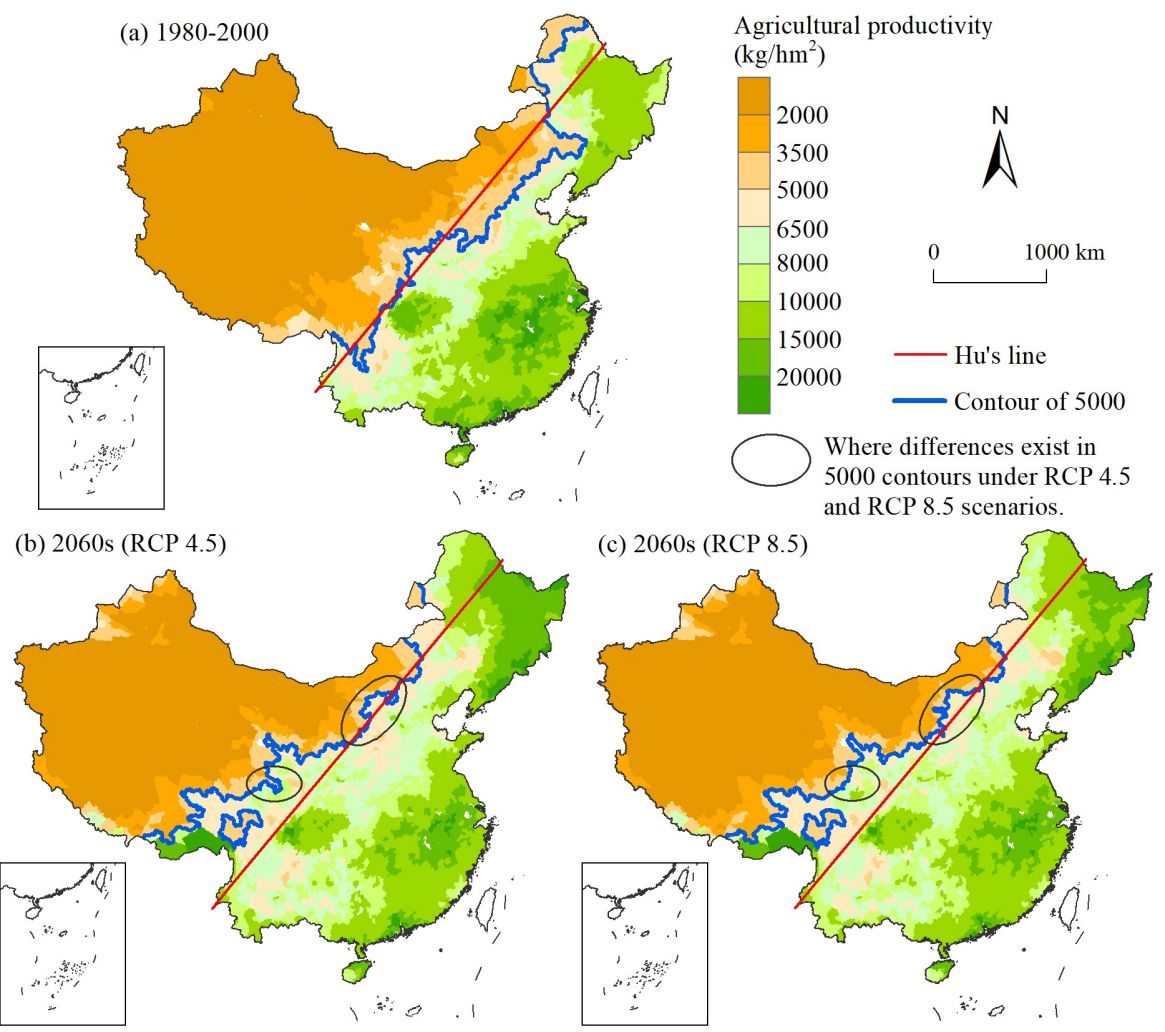
Maps of potential agricultural productivity.

The results show that the potential agricultural productivity exhibits an obvious geographical differentiation in China. In the historical phase, bounded by Hu’s line, areas east of the line have a higher potential agricultural productivity, primarily over 6500 kg/hm^2^, while productivity is principally lower than 2000 kg/hm^2^ in areas west of Hu’s line (Fig 10(A)). Hu’s line is the basic lock of potential agricultural productivity in China, and the contour of 5000 basically coincides with the line. East of the line, areas with higher potential agricultural productivity are mainly distributed in the Sichuan Basin, the middle and lower reaches of the Yangtze River, and southern and northeastern China.

In the 2060s, the spatial trends of potential agricultural productivity show little difference: high in northwestern China and low in southeastern China. However, there is a large deviation in the contour of 5000, moving to the northwest overall, especially in the southwest. This observation indicates that areas around Hu’s line represent the increasing trend of potential agricultural productivity due to the changes in water and temperature. The contour lines are similar under the two scenarios, and the obvious differences are marked with ellipses in Fig 10, 10B and 10C.

**Fig 11:**
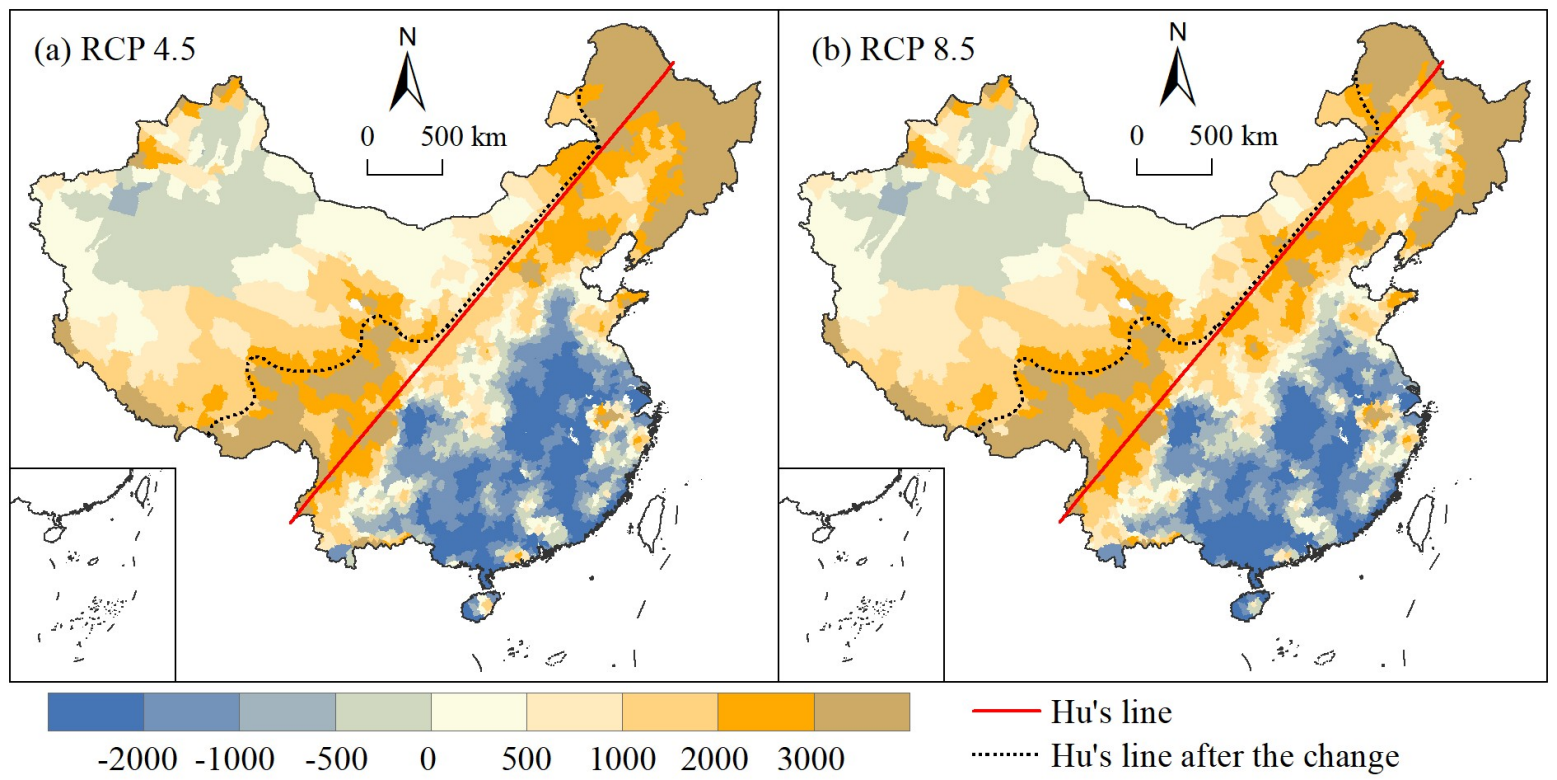
Changes in potential agricultural productivity and Hu’s line.

To highlight where the variations occur, the potential agricultural productivity during 1980–2000 is subtracted from that in the 2060s under the two scenarios. The differences between the two periods are shown in Fig 11, showing an obvious spatial differentiation characteristic. The potential agricultural productivity in the southeastern region exhibits a downward trend. In the northwestern region, rising and falling trends coexist, but the magnitude of change is relatively small. The potential agricultural productivity mainly shows an upward trend in the northeastern and southwestern regions. As a whole, areas with a large increase in the potential agricultural productivity are mainly located near Hu’s line. This finding indicates that under the background of climate change, the combined effect of water resources and temperature leads to a significant upward trend in the potential agricultural productivity in eastern Tibet, western Sichuan, northern Yunnan and northwestern Inner Mongolia. There are partial breakthroughs in Hu’s line locking potential agricultural productivity.

Comparing the changes in the potential agricultural productivity under the RCP 4.5 and 8.5 scenarios, the difference is not large in Southwest China, with an increase of more than 2000 kg/hm^2^. However, in Northeast China, areas with an increase of 2000 kg/hm^2^ under RCP 4.5 are significantly larger than those under RCP 8.5, which indicates that the RCP 4.5 scenario is beneficial to agricultural development in Northeast China. Due to more available precipitation on the Loess Plateau under the RCP 8.5 scenario, the potential agricultural productivity exhibits a greater increase compared with that under the RCP 4.5 scenario. Hu’s line does not show a clear breakthrough and has a strong agricultural lock in these areas.

## 4. Conclusions

In this paper, five global climate models from CMIP5 were used to evaluate the temporal and spatial variations in water resources in China under four RCP scenarios. Then, the potential agricultural productivity, affected by changes in water and temperature, was analysed under the RCP 4.5 and RCP 8.5 scenarios. The results show that the available precipitation is significantly lower than the precipitation under the combined action of precipitation and evapotranspiration, and the spatiotemporal characteristics of water resources under different scenarios are significantly different. For the national average, the available precipitation is the highest under the RCP 2.6 and 4.5 scenarios, followed by that under the RCP 8.5 scenario. In terms of spatial distribution, the amount of available precipitation shows a decreasing trend from southeast to northwest. Regarding the temporal change, the available precipitation under RCP 8.5 exhibits a trend of first increasing and then decreasing, while the available precipitation under the RCP 6.0 scenario shows a trend of first decreasing and then increasing. Under the RCP 2.6 and 4.5 scenarios, the available precipitation is rising, where the RCP 4.5 scenario has a higher rate of increase than that of RCP 2.6. In the context of climate change, potential agricultural productivity varies due to changes in water resources and temperature. The contour line of 5000 in the 2060s moves to the northwest, especially in the southwest. The potential agricultural productivity decreases in the southeastern region and increases in the northeastern and southwestern regions. Hu’s line has a partial breakthrough in the locking of agriculture, mainly in eastern Tibet, western Sichuan, northern Yunnan and northwestern Inner Mongolia.
